# ﻿Morphology of immature stages, biology, and systematic position of the Violet seed weevil, *Orobitiscyanea* (Linnaeus, 1758) (Curculionidae, Conoderinae, Orobitiditae, Orobitidini)

**DOI:** 10.3897/zookeys.1121.86888

**Published:** 2022-09-12

**Authors:** Rafał Gosik, Peter Sprick

**Affiliations:** 1 Department of Zoology and Nature Protection, Faculty of Biology and Biotechnology, Maria Curie–Skłodowska University, Akademicka 19, 20–033 Lublin, Poland Maria Curie–Skłodowska University Lublin Poland; 2 Curculio–Institute e.V. (CURCI), Weckenstraße 15, 30451 Hannover, Germany Curculio–Institute e.V. Hannover Germany

**Keywords:** *Blacus*, escape mechanism, life cycle, mimicry, parasitoid, thanatosis

## Abstract

The mature larva of the weevil species *Orobitiscyanea* (Linnaeus, 1758), one of only two Palaearctic members of the supertribe Orobitiditae, is re-described, while the pupa is described for the first time. The biology of this species was studied at two sites in Germany. It was reared from seed capsules of *Violacanina* L. (Violaceae), and feeding holes were observed on *V.riviniana* Rchb. Adults of *Orobitiscyanea* and *O.nigrina* Reitter, 1885, specialists of *Viola*, show a well-developed escape mechanism, to which contribute a smooth surface, a rounded, nearly spherical body shape, and a seed-imitating thanatosis behaviour. The molytine weevil *Leiosomacribrum* (Gyllenhal, 1834), the only other known weevil specialist of *Viola* in Europe, has a smooth surface, also, and is the most spherical species of the genus. The unique characters of the larva and pupa of *Orobitiscyanea* are discussed in regard to the systematic position of this taxon.

## ﻿Introduction

The subfamily Conoderinae Schoenherr, 1833, in the broad sense of some current classifications (Prena et al. 2014; Alonso-Zarazaga et al. 2017), is distributed worldwide and contains four supertribes: Bariditae Schoenherr, 1836; Ceutorhynchitae Gistel, 1848; Conoderitae Schoenherr, 1833 (also known as Zygopinae Lacordaire, 1865), and Orobitiditae C. G. Thomson, 1859. From the total number of 7571 described Conoderinae species in 940 genera, approximately 4032 belong to Bariditae, 2164 to Conoderitae, ~ 1371 to Ceutorhynchitae, and just four to Orobitiditae (Prena et al. 2014). The Violet Weevils (Orobitiditae) include two genera: 1) *Parorobitis* Korotyaev, Konstantinov & O’Brien, 2000 with two Neotropical species: *P.gibbus* Korotyaev, Konstantinov & O’Brien, 2000 and *P.minuta* Korotyaev, Konstantinov & O’Brien, 2000; 2) *Orobitis* Germar, 1817 with two Palaearctic species: *O.nigrina* Reitter, 1885 and *O.cyanea* (Linnaeus, 1758) (Korotyaev et al. 2000). Orobitiditae are a very uniform group, owing to the extraordinarily convex body, 1.8–3.5 mm in size, the rostrum bent at the antennal insertion, the fused meso- and metasternum, the first ventrite no longer than the second, the claws with appendages fused in an entire median process, and the unique structure of the stridulatory device (Lyal and King 1996; Korotyaev et al. 2000). The distribution of *Orobitiscyanea* includes Europe from the Arctic Circle to the Mediterranean, Siberia, central and east Asia with Asia Minor (Morris 2012; Alonso-Zarazaga et al. 2017). As *Orobitiscyanea* has limited dispersal abilities owing to its reduced or non-existent wings (Dieckmann 1972), it inhabits a wide range of natural or near-natural habitats: well-insolated deciduous woodlands and forest edges, nutrient-poor grasslands, limestone grasslands, marshes, sand dunes, and cliffs (Smreczyński 1974; Morris 2012). Interestingly, it has also been reported as a pest species in nurseries (Dieckmann 1972), but this latter observation, dating 50 or more years ago, could indicate that nurseries were then situated close to natural habitats or that plant material was exchanged between them.

To date, the systematic placement of Orobitidinae has been changed many times. Van Emden (1938) accepted the status of Orobitinae (sic) in the rank of a subfamily. At the same time, taking into account the morphology of the larval stage, he drew attention to their fundamental distinctiveness from Ceutorhynchinae (originally Ceutorrhynchinae), and he noticed some similarities between Orobitinae, Apioninae, and Gonatoceri. Dieckmann (1967, 1972), Lohse (1983), and Smreczyński (1974) included Orobitidinae in Ceutorhynchinae, whereas Zherikhin and Gratshev (1995) placed *Orobitis* in an enlarged concept of Barididae.

However, Alonso-Zarazaga and Lyal (1999) extracted them as a separate subfamily. Also, Korotyaev et al. (2000), based on a very detailed morphological analysis of the adult stage of Ceutorhynchinae, Zygopinae (i.e., Conoderinae sensu stricto), Baridinae, and Orobitidinae, left all these groups in the rank of subfamilies, emphasising especially the distinctiveness of Orobitidinae from the others. At the same time, they noted that the final decision about their placement required further research. The treatment of Orobitidinae as a separate subfamily was also upheld by Lyal (2013). In contrast, Prena et al. (2014) reduced this subfamily to supertribe rank within the subfamily Conoderinae, while still highlighting the significant differences (both in adults and in larvae) between Orobitidinae and the other taxa grouped in Conoderinae sensu lato. The position of Orobitidinae in the supertribe rank within Conoderinae was subsequently retained by Alonso-Zarazaga et al. (2017).

In view of the difficulty in clarifying of the taxonomic position of this widespread Palaearctic species, the critical morphological differences to other Conoderinae, some important discrepancies between previously published information on the larval stage (Urban 1925; van Emden 1938), our observations concerning the biology of the Violet seed weevil, and the lack of a description of its pupa, the purpose of this contribution is to provide new morphological information on the larval stage and to describe the pupa of the taxonomically isolated genus *Orobitis*, that may be valuable to clarify its systematic position. In his excellent paper, van Emden (1938) listed only some of the features of *Orobitis* that are different from other Conoderinae.

## ﻿Materials and methods

### ﻿Study sites

**Figure 1.**
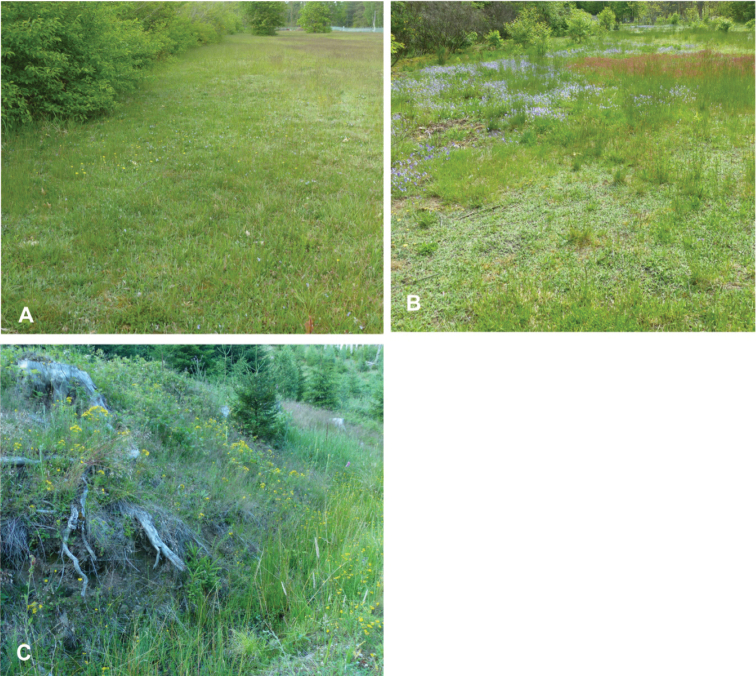
Biotopes of *Orobitiscyanea***A, B** dry, nutrient-poor grassland on sandy soil near Scheuen **C** clearing on a well-insolated south–facing slope in the Harz National Park, 470 m a.s.l.

On 3 July 2020, *Orobitiscyanea* was detected in stands of *Violacanina* L. in nutrient-poor grassland on a military training area near the village of Scheuen in the Celle district of Lower Saxony (Niedersachsen) (Fig. 1A, B). Two other *Viola* species, present at the same site and in the same habitat, were *Violaarvensis* Murr. and ViolatricolorL.subsp.tricolor. Whereas *V.arvensis* occurred mainly in small numbers, *V.tricolor* was very common at several spots there. In 2021, this site was visited once again, and the search for larvae and pupae was repeated on 19 June 2021. On 17 July 2021, the species was found in the ‘Kleines Sandtal’ (‘Small Sand Valley’) locality in the Harz National Park in the federal state of Sachsen-Anhalt, 3.6 km south-west of Ilsenburg. The specific microhabitat lays at the foot of a well-insolated south-facing slope, mainly along the border of a ditch with *Violariviniana* Rchb. (Fig. 1C). The altitude is ~ 470 m a.s.l.

### ﻿Material studied

**Figure 2.**
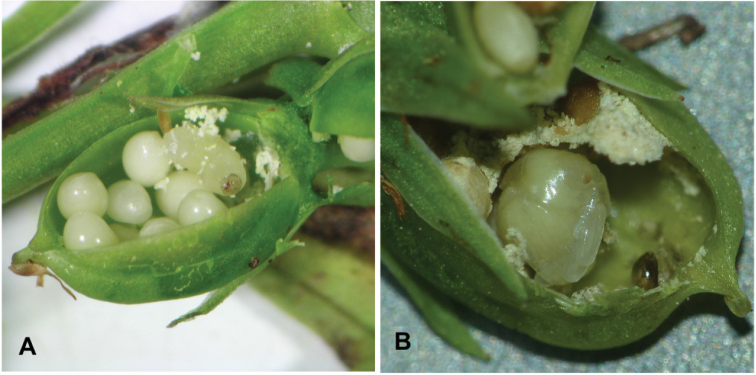
*Orobitiscyanea***A** larva and seeds in a fruit of *Violacanina***B** pupa in seed capsules of *V.canina*.

**Larvae**: 10 exx. 03.07.2020, Scheuen (Celle), military training area, dry, nutrient-poor grassland on sandy soil, in *Violacanina* seed capsules (Fig. 2A).

**Pupae** ♀: 1 ex. 03.07.2020, 2 exx. 19.06.2021, Scheuen (Celle), military training area, dry, nutrient-poor grassland on sandy soil, in *Violacanina* seed capsules (Fig. 2B).

### ﻿Methods

Before description, all the specimens were fixed in 75% ethanol and examined under an optical stereomicroscope (Olympus SZ 60 and SZ11) with calibrated oculars. The following measurements of the larva were made: body length (**BL**), body width (**BW**) (at the third thoracic segment), head capsule width (**HW**) and head capsule height (**HH**, measured from the apex to the epistoma). The pupal measurements included body length (BL), body width (BW) (at the level of the mid-legs), head width (HW) (at the level of the eyes), length of rostrum (**RL**) and width of pronotum (**PW**). Drawings and outlines were made using a drawing tube (MNR–1), installed on a stereomicroscope (Ampliwal), and were processed with computer software (Corel Photo-Paint X7, Corel Draw X7).

Slide preparation basically followed May (1994). The larva selected for study under the microscope was cut off and clear, next the mouth parts were separated. The remaining part of the body was cleared in 10% potassium hydroxide (KOH), then rinsed in distilled water and dissected. Consequently, the head, mouthparts and body (thoracic and abdominal segments) were separated and mounted on permanent microscope slides in Faure–Berlese fluid (50 g gum arabic and 45 g chloral hydrate dissolved in 80 g distilled water and 60 cm^3^ glycerol) (Hille Ris Lambers 1950).

The photographs were taken using an Olympus BX63 microscope and processed with Olympus cellSens Dimension software. The larvae selected for SEM imaging (scanning electron microscope) were first dried in absolute ethanol (99.8%), then rinsed in acetone, treated by CPD (Critical Point Drying) and finally gold-plated. TESCAN Vega 3 SEM was used to examine selected structures.

The general terminology and chaetotaxy follow Anderson (1947), May (1994), Marvaldi (1999, 2003), and Skuhrovec et al. (2015); the terminology for the antennae follows Chaika and Tomkovich (1997). Larval instar determination and calculation of the Growth Factor (GF) are based on Willis (1964) and Gosik et al. (2019).

## ﻿Results

### ﻿Description of the larva of *Orobitiscyanea*

**BL**: 1.00–4.00; BH: 0.57–1.43; HW: 0.37–0.58 (all measurements are given in mm). The detailed results of measurements and the Growth Factor calculation are listed in Table 1.

**Table 1.** Measurements and Growth Factor calculation in *Orobitiscyanea* larvae (measurements are given in mm, ^n^–number of specimens; HW is relevant to GF calculation; abbreviations: BL–body length, BW–body width, HW–head width; HH–head height).

Instar	HW	HH	BL	BH	GF
1^st^ instar	0.37^1^; 0.38^1^	0.35^1^; 0.55^1^	1.00^1^; 1.05^1^	0.57^1^; 0.60^1^	
2^nd^ instar	0.46^2^; 0.47^1^	0.40^2^; 0.42^1^	3.00^2^; 3.16^1^	0.83^2^; 1.00^1^	1.23
3^rd^ instar (mature)	0.57^2^; 0.58^3^;	0.46^2^; 0.50^2^; 0.53^1^	3.00^1^; 3.66^2^; 4.00^2^	1.00^1^; 1.16^1^; 1.33^2^; 1.43^1^;	1.24

**Figure 3.**
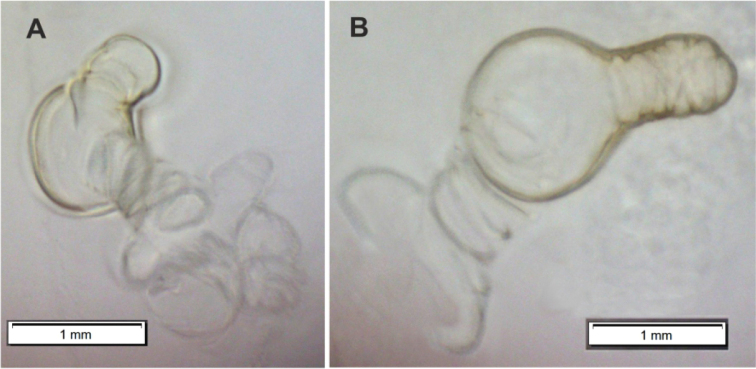
*Orobitiscyanea* mature larva, spiracles **A** spiracle of prothorax **B** spiracle of abdominal segment I.

**General habitus and chaetotaxy.** Live larva pure white, with yellow head capsule (Fig. 2A). All spiracles unicameral; thoracic (Fig. 3A) placed laterally between pro- and mesothorax; abdominal spiracles (Fig. 3B) placed medio-laterally on segments I–VIII. Body rather elongate, curved, rounded in cross section.

**Figure 4.**
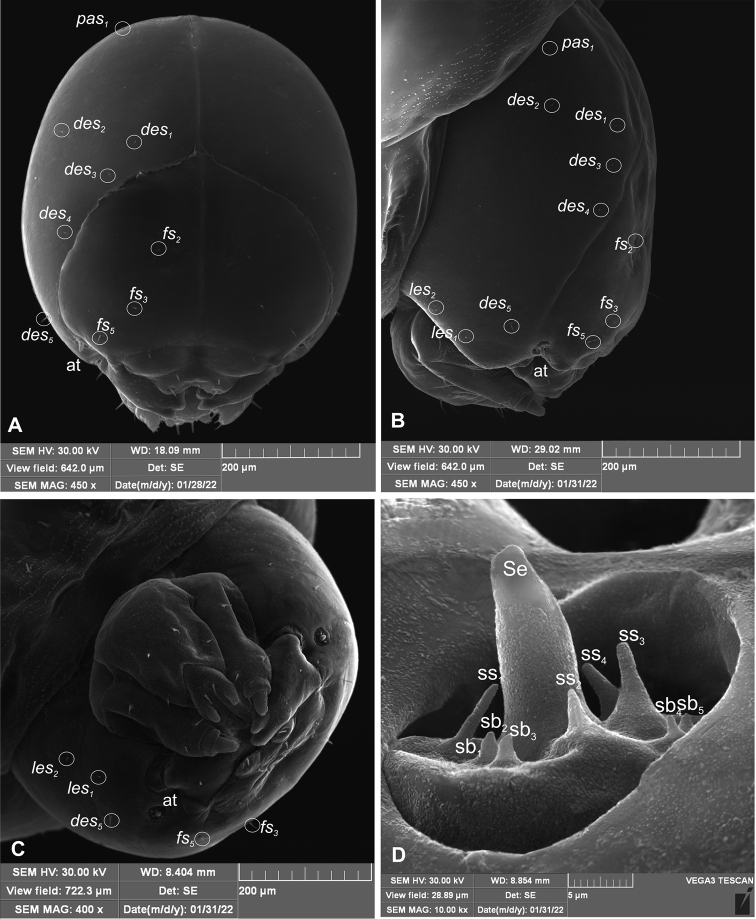
*Orobitiscyanea* mature larva, head and antenna (SEM micrograph) **A** frontal view **B** lateral view **C** ventral view **D** antenna. Abbreviations: at – antenna, sb – sensillum basiconicum, Se – sensorium, ss – sensillum styloconicum, setae: *des* – dorsal epicranial, *fs* – frontal, *les* – lateral epicranial, *pes* – postepicranial.

**Head and antenna.** Head capsule (Fig. 4A–C) almost rounded; endocarina reaches 4/5 of the frons; frontal sutures distinct along entire length up to antennae; stemmata (st) invisible. Hypopharyngeal bracon without median sclerome. Setae of head minute, only *des_5_* and setae on frons short, hair–like. Cranial setae: *des_1_* placed medially, *des_2_* placed posterolaterally, *des_3_* and *des_4_* placed suture on epicranium away from frontal suture, *des_5_* placed anterolaterally, *fs_2_* placed medially, *fs_3_* placed anteromedially, *fs_5_* placed anterolaterally, close to epistome, *les_1_* and *les_2_* placed close to *des_5_*, postepicranial area with one *pes.* Antennae (Fig. 4D) placed on each side at anterior margin of head; membranous basal segment convex, semi–spherical, bearing conical, distinctly elongated sensorium and nine sensilla: five basiconica (sb) and three styloconica (ss).

**Figure 5.**
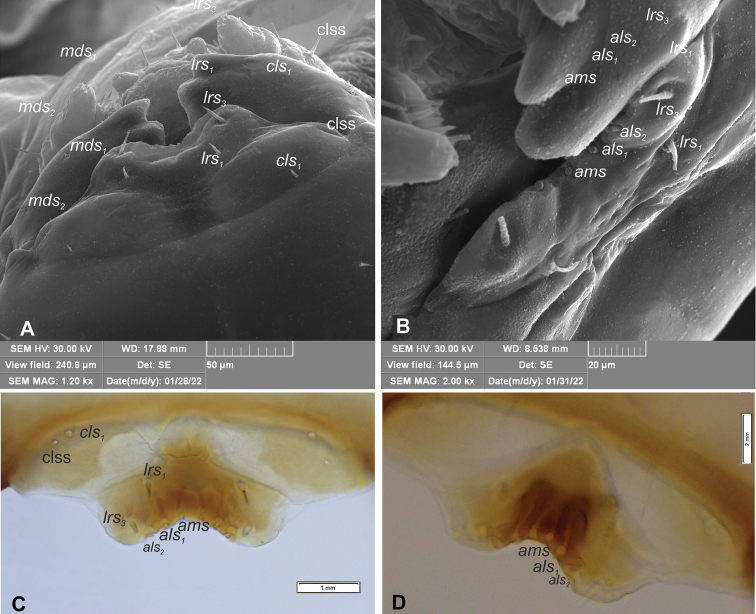
*Orobitiscyanea* mature larva, clypeus, labrum, epipharynx and mandible **A, B** clypeus and labrum (SEM micrographs) **C** clypeus, labrum and epipharynx **D** epipharynx with ribs. Abbreviations: clss – clypeal sensorium, setae: *ams* – anteromedial, *als* – anterolateral, *cls* – clypeal, *lrs* – labral.

**Figure 6.**
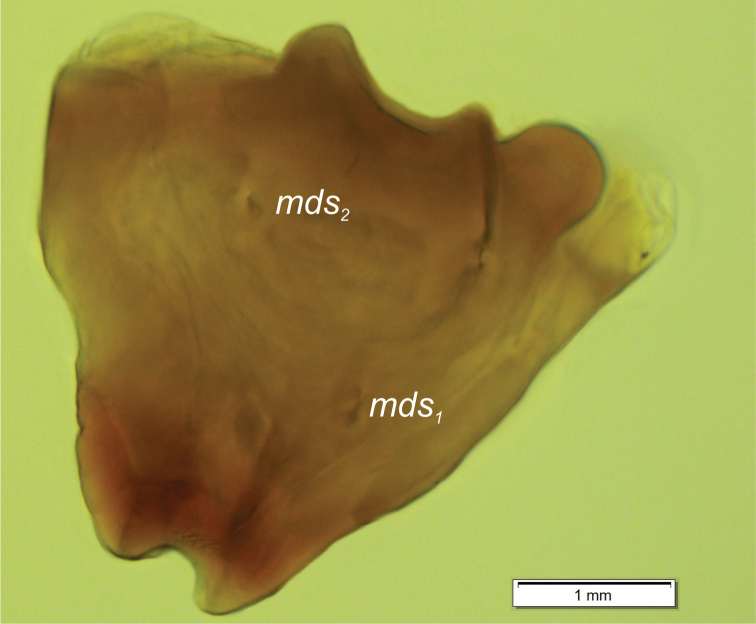
*Orobitiscyanea* mature larva, left mandible. Abbreviation: *mds* – mandibular setae.

**Figure 7.**
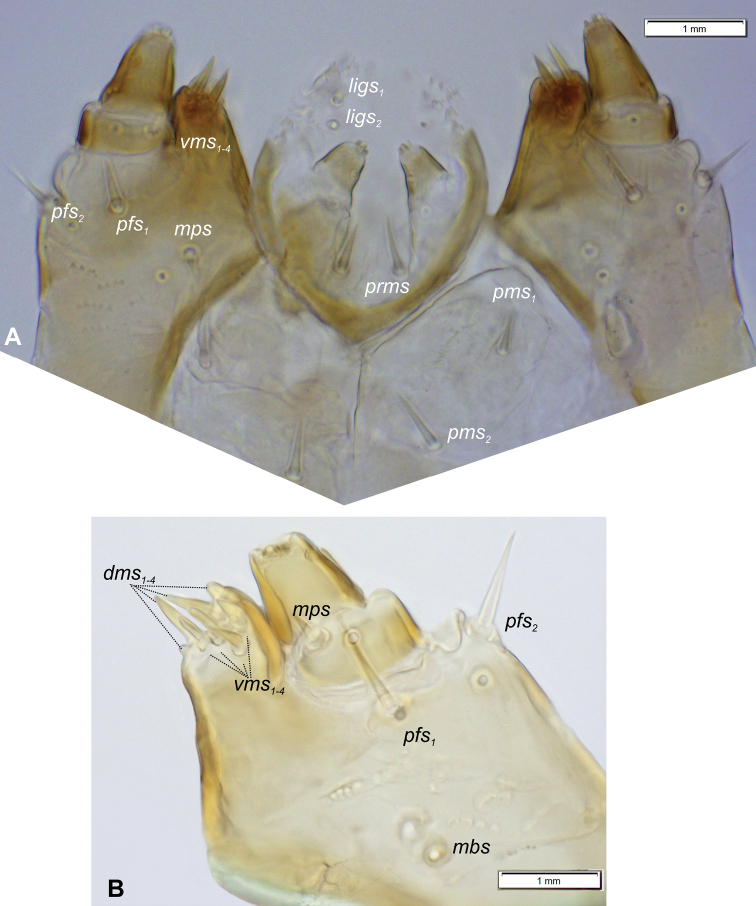
*Orobitiscyanea* mature larva, maxillolabial complex and apical part of maxilla **A** maxillolabial complex, ventral aspect **B** apical part of left maxilla, photo. Abbreviations: setae: *dms* – dorsal malar, *ligs* – ligular, *mbs* – malar basiventral, *mps* – maxillary palp, *pfs* – palpiferal, *prms* – prelabial, *pms* – postlabial, *stps* – stipal, *vms* – ventral malar.

**Figure 8.**
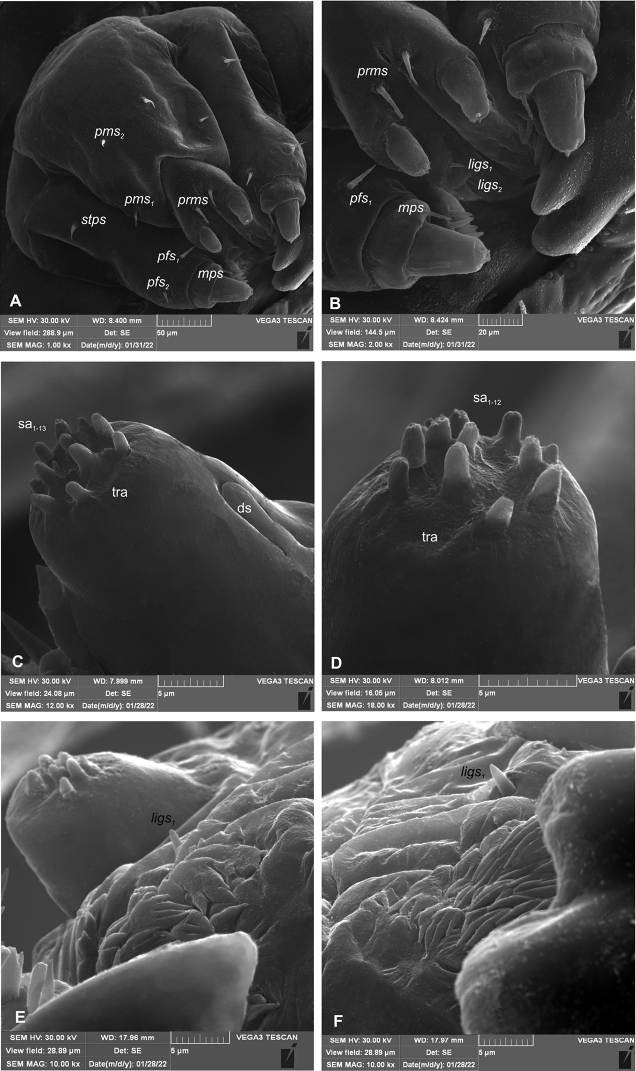
*Orobitiscyanea* mature larva, maxillolabial complex (SEM micrographs) **A** maxillolabial complex, ventral aspect **B** prementum, ventral aspect **C** apical part of distal maxillary palp **D** apical part of labial palpomere **E, F** surface of ligulae. Abbreviations: ds–digitiform sensillum, sa – sensillum ampullaceum, tra – terminal receptive area, setae: *dms* – dorsal malar, *ligs* – ligular, *mbs* – malar basiventral, *mps* – maxillary palp, *pfs* – palpiferal, *prms* – prelabial, *pms* – postlabial, *stps* – stipal, *vms* – ventral malar.

**Mouthparts.** Clypeus (Fig. 5A) ~ 4.5× wider than long, with single *cls* medium in size, placed posteromedially, sensillum (clss) posterolaterally. Anterior margin of clypeus distinctly concave. Labrum (Fig. 5A, B) ~ 2× wider than long, anterior margin sinuated; *lrs_1_* medium, placed anteromedially, *lrs_2_* absent and *lrs_3_* medium, placed posterolaterally. Epipharynx (Fig. 5C) with two *als* and one *ams*, all semi-circular, *mes* absent. Labral rods (lr) absent as such but five sclerotisations like ribs distinct between the *ams* and *als* (Fig. 5D). Clypeus and labrum distinct, with transverse, median furrow. Mandible (Fig. 6) with two apical teeth of almost equal height, the inner one subapical and slightly smaller; cutting edge smooth, without additional protuberance; setae: *mds_1_* and *mds_2_* minute, both placed medially in shallow pits. Maxillolabial complex: (Figs 7A, B, 8A) stipes with a medium *stps*, two short *pfs*, and one minute *mbs* plus sensillum; mala with row of four *dms* various in shape and size (first semi-circular, second and third elongated, pointed, fourth short, blunt) and a group of four digitate, medium *vms*; maxillary palpi bi-segmented; basal palpomere distinctly wider and shorter than distal one; length ratio of basal and distal palpomeres 2:1; basal palpomere with medium short *mps* and one pore, distal palpomere (Fig. 8B) with one digitiform sensillum (ds) and a group of 13 apical sensilla (ampullacea) on terminal receptive area (tra) (Fig. 8C); dorsal parts of mala partially covered with fine asperities; labium with cup-shaped prementum, with one medium *prms* placed medially (Fig. 7A); ligula divided, with two minute *ligs*, at margin covered with prominent asperities (Fig. 8B, E, F); premental sclerite C-shaped; postmentum rather elongate, and narrow, membranous, triangular, with two medium *pms: pms_1_* situated posterolaterally and *pms_2_* mediolaterally; labial palpi one–segmented; each palpus with single pore, distal palpomere with a group of 12 apical sensilla (ampullacea) on terminal receptive area (Fig. 8D); surface of labium smooth.

**Figure 9.**
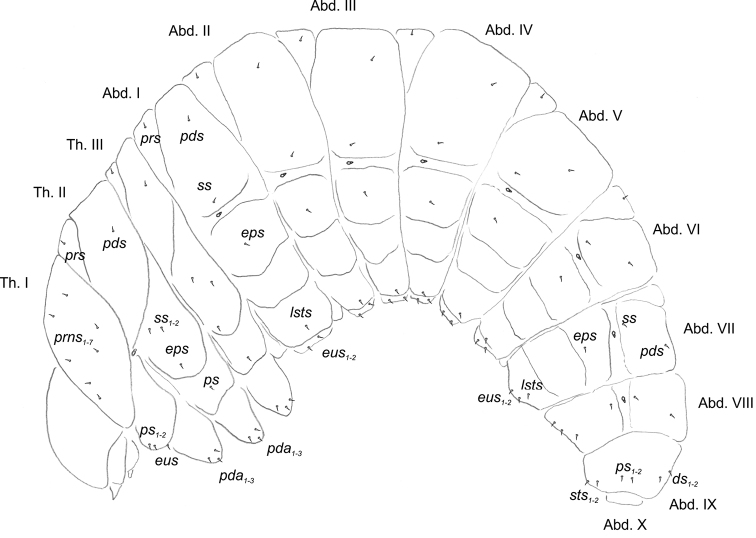
*Orobitiscyanea* mature larva, lateral view, habitus and chaetotaxy. Abbreviations: Th. I–III–thoracic segments 1–3, Abd. I–X–abdominal segments 1–10, setae: *ds*–dorsal *eps*–epipleural, *eus*–eusternal, *ps*–pleural, *pda*–pedal, *pds*–postdorsal, *prns*–pronotal, *prs*–prodorsal, *ss*–spiracular, *sts*–sternal.

**Figure 10.**
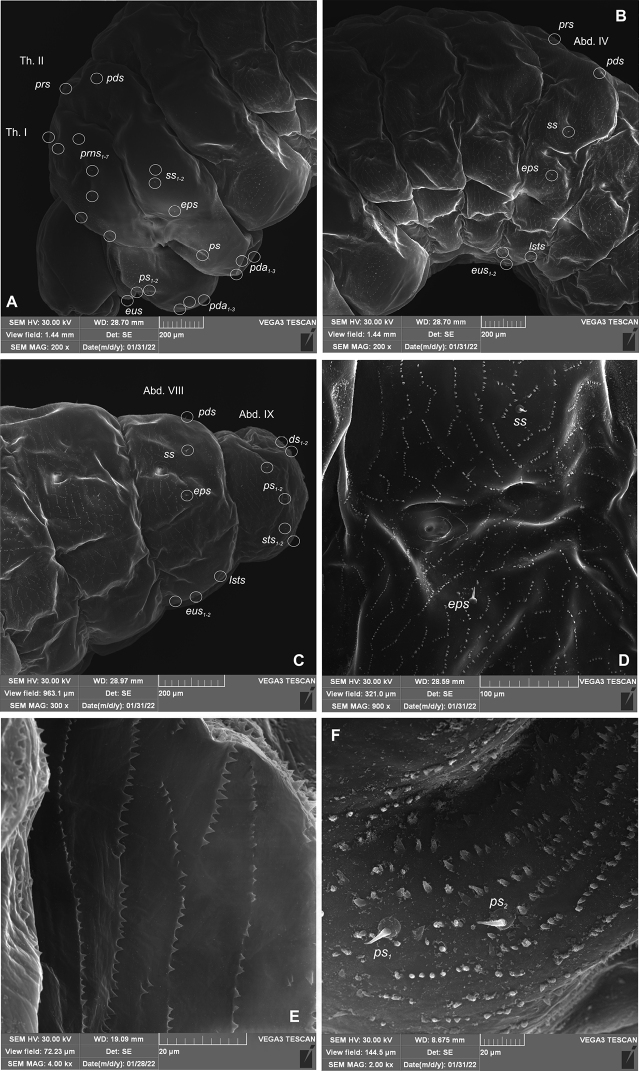
*Orobitiscyanea* mature larva, habitus and cuticle (SEM micrographs) **A** lateral view of head and thorax **B** lateral view of abdominal segments I–V **C** lateral view of abdominal segments VII–IX **D** lateral view of abdominal segment V (magnification) **E** structure of cuticle of dorsolateral part of prodorsum **F** structure of cuticle of ventrolateral part of prodorsum. Abbreviations: setae: *ds*–dorsal *eps*–epipleural, *eus*–eusternal, *ps*–pleural, *pda*–pedal, *pds*–postdorsal, *prns*–pronotal, *prs*–prodorsal, *ss*–spiracular, *sts*–sternal.

**Body.** Prothorax small, pronotal shield not pigmented; mesothorax slightly smaller than metathorax. Meso- and metathorax each divided dorsally into two lobes (prodorsal and postdorsal lobes almost equal in size). Pedal lobes of thoracic segments isolated, conical, prominent. Abdominal segments I–III of similar size, slightly smaller than metathorax (Figs 9, 10A). Segments IV–IX tapering towards posterior body end. Abdominal segments I–VII each with weakly developed prodorsal fold and prominent, undivided postdorsal lobe (Figs 9, 10B). Segments VIII–IX dorsally undivided. Epipleural lobes of segments I–VII slightly conical, on segments VIII and IX almost invisible. Laterosternal and eusternal lobes of segments I–VIII conical, weakly isolated (Figs 9, 10C). Abdominal segment X divided into three lobes, dorsal small, lateral lobes prominent, of almost equal size. Anus situated terminally. Body cuticle with asperities forming rows and circles (Fig. 9D). Lateral part of prothorax densely covered with thorn-like asperities, arranged in vertical rows (Fig. 9E, F).

**Chaetotaxy**: distinctly reduced, most setae minute, thorn–like, only on dorsal part of abdominal segment IX very short, hair–like. Thorax (Fig. 9A): prothorax with seven equal in size *prns*, two *ps*, and one *eus.* Meso- and metathorax each with one *prs* and one *pds*, two *ss*, one *eps*, one *ps* and one *eus.* Pedal areas of thoracic segments each with three *pda.* Abdomen (Fig. 9B, C): segments I–VI with one *prs*, one *pds*, one *ss*, one *eps*, one *lsts*, and one *eus.* Abdominal segments VII and VIII with one *pds*, one *ss*, one *eps*, one *lsts*, and two *eus.* Abdominal segment IX with two *ds*, two *ps*, and two *sts.* Abdominal segment X without setae.

### ﻿Description of the pupa of *Orobitiscyanea*

**Female**: BL: 2.00^1^; 2.16^1^; 2.20^1^; BW: 2.16^1^; 2.33^1^; HW: 0.55^1^; 0.57^2^; RL: 1.00^1^; 1.05^1^; 1.10^1^; PW: 1.23^1^; 1.30^1^ (one pupa partially deformed). ^n^–number of specimens.

**Figure 11.**
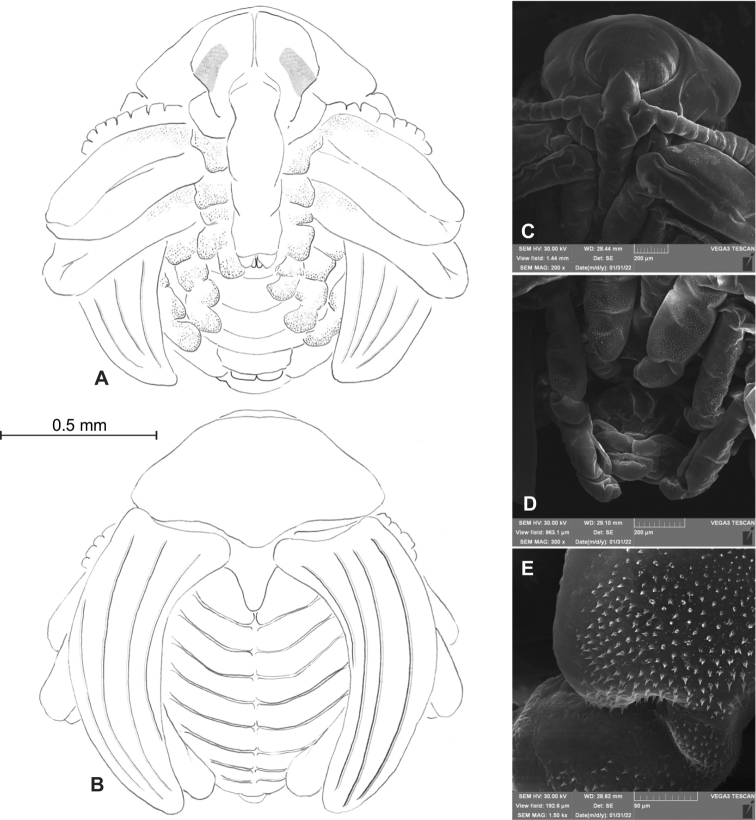
*Orobitiscyanea* pupa, habitus and structure of cuticle **A** ventral view **B** dorsal view, scheme **C** head and rostrum, frontal view **D** abdomen, ventral view **E** tarsi of first pairs of fore legs, magnification (SEM micrographs).

**General habitus and chaetotaxy.** Body white, compact, almost round in outline (Figs 2B, 11A, B), partially (femora and tarsi) covered with fine asperities, rest of body smooth (Fig. 11C–E). Rostrum elongate, almost 4× as long as wide, reaching metacoxae. Pronotum trapezoidal, 2× wider than long. Mesonotum wider than metanotum, with prominent triangular scutellar shield. Abdominal segments I–V of equal length, segments VI–VIII tapering gradually towards end of body, segment IX terminal. Gonotheca in female divided. Urogomphi (posterior processes) absent. Spiracles placed laterally on abdominal segments I–VI, functional on segments I–V, vestigial on segment VI. Chaetotaxy completely reduced, invisible even under the highest magnification.

### ﻿Biological observations on host plants, life cycle, and antagonists of *Orobitiscyanea*

**Figure 12.**
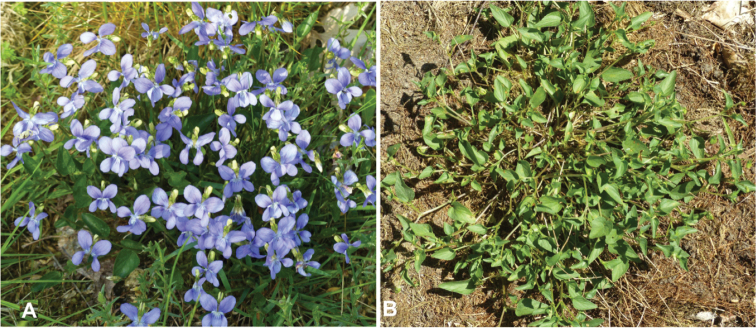
*Violacanina***A** flowering host plant **B** host plant with some feeding holes after flowering.

**Figure 13.**
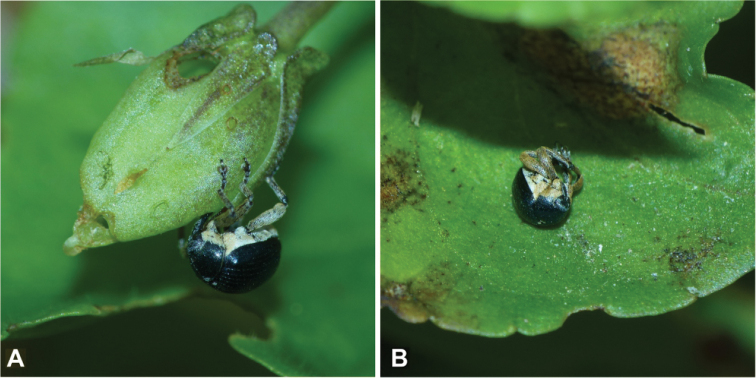
*Orobitiscyanea* on *Violariviniana***A** adult on seed capsule with feeding traces and emergence hole **B** adult exhibiting thanatosis.

A search for immature stages at the site near Scheuen yielded several larvae and a few pupae on 19 June 2020 and 3 July 2021. They were found only in seed capsules of *Violacanina* (Fig. 12A). The examination of more than 20 capsules of *V.tricolor* did not reveal a single immature specimen or any feeding traces similar to those seen on *V.canina* leaves (Fig. 12B). Likewise, we did not obtain any larva or pupa at the Harz Mountains site on 17 July, but from the numerous feeding traces on the leaves and seed capsules and from the emergence holes in the capsules, the conclusion was drawn that development must have taken place in *V.riviniana* seed capsules, too (Fig. 13A, B).

In April and May, overwintering adults make small feeding holes in the leaves of their host plants, eating for maturation. At dry sites with early-flowering *Viola* species, such as *V.canina* L. or *V.hirta* L., eggs are laid mainly in April and May in the immature ovaries of the flowers. Larvae feed from young seeds, generating sufficient room to develop into the pupal stage at their feeding sites. Pupation occurred at both study sites inside the seed capsules in June and the first half of July. Adults left the seed capsules actively through feeding holes, or at the latest in July, by which time the seeds had ripened and the seed capsules burst open. Even in the Harz Mountains, the new generation had totally abandoned its place of development at the well-insolated site by mid-July, and some individuals were now occurring on their host plants; at that time, many adult weevils were present only in less exposed places. There were many feeding traces and adult weevils on the plants, but there were no more larvae or pupae inside the seed capsules. These were either still closed along the shady trench or had burst open on the sun-exposed slope.

## ﻿Discussion

### ﻿Comments and inferences regarding the host plants, biology and parasitoids of *Orobitiscyanea*

Our observations regarding the feeding and development of *Orobitiscyanea* on *Violariviniana* seed capsules are in accordance with those of Scherf (1964), who listed the following *Viola* species as host plants of this weevil: *V.canina*, *V.epipsila* Ledeb., *V.odorata* L., *V.palustris* L., *V.reichenbachiana* Jord. ex Bor. (as *V.silvatica* Fr. ex Hartm.) and *V.riviniana*. In addition, Urban (1925) listed *Violacanina* (once under this name and once as *V.stricta* Hornem.), *V.odorata*, *V.palustris*, *V.pumila* Chaix (as *V.pratensis* Mert. & Koch), and *V.reichenbachiana* Jord. ex Bor. (as *V.silvestris* auct.). The only species to add from our own observations is *V.hirta* L., from which *O.cyanea* was swept on one occasion in Luxembourg. But as this was only a singular finding without observation of feeding traces, the host plant status of *V.hirta* for *O.cyanea* has still to be confirmed. All these *Viola* species are violet in colour, and none are pansy species like *V.arvensis*, *V.tricolor* or *V.×wittrockiana* Gams ex Neuenb. & Buttl., which are also members of the genus *Viola*. Dieckmann (1972) described the development of *O.cyanea* in seed capsules, but he did not list any particular *Viola* species. His statement that *O.cyanea* was found to be a pest on pansy species has therefore to be regarded as doubtful. According to our observations, *O.cyanea* is related to natural habitats, but Morris (2012) reported it also from cultivated areas like pastures and gardens.

On the other hand, we confirm the information given by Dieckmann (1972) regarding the phenology and pupation of *Orobitiscyanea* in a cocoon, in the immediate vicinity of the feeding place or directly there. Our observations are contrary to those of Urban (1925), who reported a late start of development in the season, and violet seed capsules with larvae, pupae, fresh and fully coloured adults of *O.cyanea* that were studied in September. As Urban (1925) did not supply any data on either site or host plants, it can only be assumed that he studied the development of this weevil in moist, shady or cool sites with late-flowering host plant species, such as *Violaepipsila* or *V.palustris.* There, the first adults of the new generation should occur considerably later, in July and August, and the latest adults may hibernate at the pupation sites inside the capsules. Obviously, the activity of this oligophagous weevil is closely linked to plant development and may even differ from one locality to another as a result of microclimatic differences, as observed at the study site in the Harz Mts.

**Figure 14.**
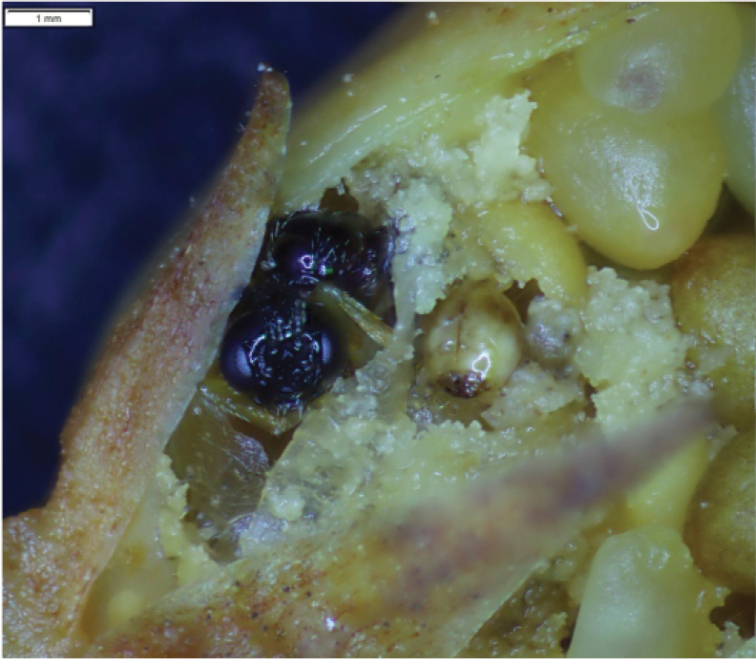
*Orobitiscyanea* larva and the parasitoid wasp.

Some specimens of a parasitoid wasp, *Blacus* sp. (Fig. 14), were found in the samples of seed capsules containing *Orobitiscyanea* larvae from Scheuen, taken to the laboratory for rearing. Wasps of the genus *Blacus* Nees, 1818 (Blacini, Braconidae) are common parasitoids of weevil larvae and are frequently reported from weevil genera like *Scolytus*, *Stereonychus*, *Gymnetron* and *Barynotus* (Belokobylskii 1995; Farahani and Talebi 2013).

### ﻿Larval instar determination and Growth Factor calculation

The method of larval instar determination worked out by Dyar (1890) has been widely accepted (Leibee et al. 1980; Rowe and Kok 1985). It was ultimately popularised under the name of Growth Factor (GF) (Sprick and Gosik 2014; Gosik et al. 2019), which is in fact Dyar’s ratio^–1^, which bears a closer relationship with natural development. The results of measurements and the Growth Factor calculation indicates three larval instars in *Orobitiscyanea* (Table 1) and GF values of 1.23 and 1.24 from the first to the second, and from the second to the third instar, respectively.

The number of larval instars in weevils is correlated primarily with the body size of a species. Thus, small species (head width of the mature larva below ~ 0.65 mm) usually have only three larval instars (Dosdall et al. 2007; Gosik et al. 2020; Skuhrovec et al. 2022), whereas larger species can have up to seven instars (Skuhrovec et al. 2022).

In some previously studied Entiminae species, GF usually varied between 1.38 and 1.44 (Leibee et al. 1980; Sprick et al. 2022). There is only one GF value each for a Ceutorhynchinae and Lixinae species: in *Ceutorhynchussubpubescens* LeConte, 1876 it was calculated at 1.43 (Dosdall et al. 2007) and in *Rhinocyllusconicus* (Frölich, 1792) at 1.538 (Rowe and Kok 1985). In the case of *Orobitiscyanea*, the significantly low GF value is probably correlated with the relatively big size of the first larval instar. There is usually only one larva per seed capsule (Urban 1925). Taking into consideration the limited dispersion capability and the close host plant affinities of *O.cyanea*, females invest more in the size than in the number of eggs.

### ﻿Morphological adaptations and behaviour

When disturbed, *Orobitiscyanea* shows death-feigning or thanatosis behaviour and appears to imitate a *Viola* seed, which may be a form of mimicry: the dark part of the weevil may imitate the main part of the seed, the light part the elaiosome (Morris 2012; Kutzelnigg 2013). Thanatosis, the spherical shape and the colouring prove to be effective components of a shelter mechanism when escaping from danger, as they allow rapid down-rolling and concealment in vegetation or leaf litter below the plants, possibly among seeds or dark soil particles at the same time (Fig. 13B), especially as the smooth leaves of most *Viola* species support this escape mechanism. In addition, the behavioural data relating to *Orobitiscyanea* are presumably applicable to the closely related *O.nigrina* Reitter, 1885, which lives on *Violabiflora* L. in the Alps (Penecke 1922). Escape mechanisms similar to those described here for *Orobitis* species from *Viola* are widespread in weevils or even other beetles. Apparently, they have developed independently many times.

It seems worth mentioning that the only other European *Viola*-inhabiting weevil specialist, *Leiosomacribrum* (Molytinae), occupies the top position in spherical body shape among all available *Leiosoma* species, where this could be tested (Table 2). In 21 of ~ 44 *Leiosoma* species, available as adult specimen or as habitus photo from different sources, e.g., Pedroni (2010, 2012) or Bahr (2021), we determined the length-width ratio of the body to demonstrate the degree of spherical body shape (Table 2).

**Table 2.** Comparison of length-width ratio of the body of *Viola*-inhabiting species (A) and from species with other or unknown host plants (B). Measured from the front margin of the eyes to the apex of elytra and at the widest part of the elytra. *Groups defined by Pedroni (2010, 2012, 2018).

Host	Species/Species group*	Ratio	Data source
A	*Orobitiscyanea*	1.51:1	own data
	*O.nigrina*	1.58:1	own data
	*Leiosomacribrum*	1.72:1	own data
B	*Leiosomacribrum* group (five further species)	1.99:1 – 2.24:1	Pedroni (2018) Bahr (2021)
	*Leiosomaoblongulum* group (four species)	2.00:1 – 2.16:1	Pedroni (2010), Diotti and Caldara (2017, 2020); own data
	*Leiosomascrobiferum* group (six species)	2.22:1 – 2.43:1	Pedroni (2012)
	Species from undefined species groups: *L.apionides*, *L.bosnicum*, *L.deflexum*, *L.kirschii*, *L.reitteri*	1.88:1 – 2.28:1	Stüben (2011), Sabaljuev (2013), Pedroni (2016), own data (coll. J. Messutat, coll. M. Stern)

*Leiosomacribrum* is the most rounded, shortest, and smallest species of this genus, approximating mostly the nearly perfect spherical shape of both *Orobitis* species. All other *Leiosoma* species are more elongate, closest are *L.reitteri* Bedel, 1884, *L.apionides* (Wollaston, 1864) (both ~ 1.88:1), and *L.deflexum* (Panzer, 1795) (1.91:1). The bulk of the species ranges between ~ 2.00:1 in *L.diottii* Pedroni, 2018, *L.osellai* Diotti & Caldara, 2020, and *L.senex* Pedroni, 2018, and 2.42 – 2.43:1 in *L.hernicum* Pedroni, 2012 and *L.komovicum* Pedroni, 2018.

Even if only for a small part of *Leiosoma* species the host plant species are known (Sprick and Krämer-Klement, in press), it is noticeable that there should be some selection pressure to weevil specialists that live on *Viola* species, which are unable to fly, to improve the escape mechanisms by falling down, rolling away or imitate biotic or abiotic structures of the environment in which they live, e.g., seed in *Orobitis*, or soil or underground in *Leiosoma*. Night activity of *Leiosoma* species may be another behavioural adaptation to reduce the possible loss of adult weevils by unspecific predators.

### ﻿The taxonomic placement of Orobitiditae - based on morphological studies of immature stages

In his comprehensive work, van Emden (1938) omitted several notable features that are probably almost impossible to notice in first-instar larvae, especially if only a light microscope is available. Above all, these are the deeply divided ligula, the T-shaped anus, the extreme reduction in head bristle size, the lack of stemmata, and the clypeus divided by a transverse furrow.

The larva of *Orobitiscyanea* is easily recognised by the following features: 1) postdorsal folds of abdominal segments I–VII undivided; 2) abdominal segments VIII and IX without prodorsal folds; 3) anus T-shaped, with dorsal and lateral lobes; 4) body cuticle with asperities forming rows and circles; 5) all spiracles unicameral; 6) epicranial setae minute; 7) stemmata absent; 8) extremely elongate endocarina, almost reaching the epistome; 9) antennal sensorium elongate; 10) clypeus with prominent median depression and curved to inside anterior margin; 11) labrum extremely narrow with anterior margin deeply rounded inwards (concave); 12) clypeus with only one pair of *cls*; 13) labrum with two pairs of *lrm*; 14) labrum with one pair of *ams*, two pairs of *als* but no *mes*; 15) labral rods absent but presence of multiple rib like sclerotisations; 16) postlabium with two pairs of *pms*; 17) labial palpi uni-segmented; and 18) ligula divided.

Knowledge of the immatures of the various Conoderinae supertribes is uneven. This is mainly because the supertribes of this subfamily differ in species numbers, distribution, individual abundance and economic importance. Bariditae and Ceutorhynchitae have been relatively well studied (Scherf 1964; Pakaluk 1993, 1994; Prena et al. 2014). Conoderitae, on the other hand, have a rather small number of species with the preimaginal stages described (Gosik et al. 2021). Nevertheless, the available material is sufficient to discover the characteristics of each group. Since the larva of *Orobitiscyanea* is the only one in the genus *Orobitis* described so far and there are only two genera in the entire supertribe Orobitiditae, the above-mentioned features can be considered diagnostic of this suprageneric taxon, although it may change with further studies.

Finding features common to all known larvae that would be diagnostic of Conoderinae sensu lato is not possible. Some larval characteristics present in all the supertribes belonging to this subfamily are in fact common to the family Curculionidae (May 1994), and then cannot be diagnostic of such a subfamily, such as 1) the numbers of *des* and *les*; 2) the numbers of some thoracic setae, i.e., two *ps*, one *eus*, one *prs*, one *eps*, one *ps*; 3) the numbers of some abdominal setae: one *lsts*, two *eus*, two *ds*, two *sts*, two *ps*; and 4) the numbers of mandibular and maxillary setae. Even though larvae from the subfamily Conoderinae Schoenherr, 1833 constitute a group that is morphologically very diverse, it is still possible to find larval features common to all of them, so long as Orobitiditae are excluded. But again, these common features are typical of the family Curculionidae: 1) abdominal segments I–VII with well–separated prodorsal folds and always divided postdorsal folds; 2) anal lobes divided into four X-shaped lobes; 3) thoracic and abdominal spiracles bicameral; 4) epicranial setae elongate; 5) endocarina absent or extending to mid-length of frons; 6) ocelli present; 7) antennal sensorium short, conical; 8) clypeus trapezium-shaped with two pairs of *cls*; 9) labrum semi-circular with rounded or slightly sinuate anterior margin, always with three pairs of *lrs*; 10) labral rods elongate, well visible; 11) labrum with three pairs of *ams*, three pairs of *als* and two *mes*; 12) labial palpi bi-segmented; 13) postlabium with three pairs of *lrm*; and 14) premental sclerite tridental, with elongate posterior extension.

In addition, the GF measurements indicate that there are three larval stages in *Orobitiscyanea*, as in Ceutorhynchitae (Scherf 1964; Dosdall et al. 2007), but not in Conoderitae (5 instars) (Gosik et al. 2021).

The features given by Prena et al. (2014) as characteristic of Conoderinae pupae are very general and are widespread in other weevil subfamilies, also. Among those mentioned by these authors, there is not a single feature unique to Conoderinae. Moreover, many of the cited features are additionally annotated “present or absent”.

In the pupae, the differences between Orobitiditae and other Conoderinae are more clearly visible: setae on head, rostrum, pronotum and abdomen always clearly visible vs no setae entirely; pupal urogomphi, which are more or less developed or reduced in Conoderinae, are completely absent in Orobitiditae.

In general, endophagous larvae have significantly shorter segmental setae than exophagous larvae (Gosik et al. 2016). However, in the case of the head bristles, the difference between the two groups is not so obvious. On the other hand, one of the characteristics of *Orobitiscyanea* larvae is the complete absence of long and medium–length setae on both body and head. The longest bristles are considered at best to be microsetae. The others are almost indistinguishable from cuticular asperities.

It is worth noting that a structure similar to “ligula with depression in middle” has been described as characteristic of the larva of only one species of Baridini, namely *Aulacobarisjohanni* (Korotyaev, 1988) (Nikulina 2013). However, it is difficult to draw any further conclusions about the relationship between these species from this feature.

Both larva and pupa of *Orobitiscyanea* display many diagnostic features and at the same time differences from other Conoderinae species that it is difficult to find arguments supporting the current systematic position of this species. We consider, therefore, that there is ample justification for retaining Orobitiditae as a separate subfamily (as suggested by Korotyaev et al. 2000).

The study of immatures of the two Neotropical Orobitiditae species could well provide new data, but at this stage, the placement of Orobitiditae within an enlarged concept of Conoderinae is not supported. Finding features unique to immatures of *Orobitis* is rather easy, but associating them with any other Curculionidae group is problematic. Therefore, leaving Orobitiditae as the subfamily Orobitidinae, as suggested by Alonso-Zarazaga and Lyal (1999) and Korotyaev et al. (2000), is best supported by our results.

## References

[B1] Alonso-Zarazaga MA Lyal CHC (1999) A world catalogue of families and genera of Curculionoidea (Insecta: Coleoptera) (excepting Scolytidae and Platypodidae). Entomopraxis, SCP, Barcelona, 315 pp.

[B2] Alonso-Zarazaga MA Barrios H Borovec R Bouchard P Caldara R Colonnelli E Gültekin L Hlaváč P Korotyaev B Lyal CHC Machado A Meregalli M Pierotti H Ren L Sánchez–Ruiz M Sforzi A Silfverberg H Skuhrovec J Trýzna M Velázquez de Castro AJ Yunakov NN (2017) Cooperative Catalogue of Palaearctic Coleoptera. Curculionoidea. Sociedad Entomológica Aragonesa, Monografias electrónicas SEA, 8, November 10: 2017, 729 pp. www.sea–entomologia.org

[B3] Anderson WH (1947) A terminology for the anatomical characters useful in the taxonomy of weevil larvae. Proceedings of the Entomological Society of Washington 49: 123–132.

[B4] Bahr F (2021) Fotokatalog ausgewählter Rüsselkäfer der paläarktischen Region. / Le Charançon No. 5. www.curci.de [accessed on 26 April 2022]

[B5] Belokobylskii SA (1995) New and rare species of the genus *Blacus* (Hymenoptera: Braconidae) from the Russian Far East. European Journal of Entomology 92: 449–467.

[B6] Chaika SYu Tomkovich KP (1997) Sensory organs of weevils (Coleoptera, Curculionidae). Entomological Review 4: 486–496.

[B7] Dieckmann L (1967) Zur Gattung *Orobitis* Germar. Entomologische Blätter 63: 50–54.

[B8] Dieckmann L (1972) Beiträge zur Insektenfauna der DDR: Coleoptera – Curculionidae: Ceutorhynchinae. Beiträge zur Entomologie 22: 3–128.

[B9] Diotti L Caldara R (2017) *Leiosomamatesiense* n. sp. dei Monti del Matese (Coleoptera, Curculionidae, Molytinae). Giornale Italiano di Entomologia 14(62): 647–650.

[B10] Diotti L Caldara R (2020) *Leiosomaosellai* n. sp. dei Monti del Cilento (Coleoptera, Curculionidae, Molytinae). Giornale Italiano di Entomologia 15(64): 711–714.

[B11] Dosdall LM Ulmer BJ Bouchard P (2007) Life History, Larval Morphology, and Nearctic Distribution of *Ceutorhynchussubpubescens* (Coleoptera: Curculionidae). Annals of the Entomological Society of America 2(2): 178–186. 10.1603/0013-8746(2007)100[178:LHLMAN]2.0.CO;2

[B12] Dyar HG (1890) The number of molts of lepidopterous larvae. Psyche (Cambridge, Massachusetts) 5(175–176): 420–422. 10.1155/1890/23871

[B13] Farahani S Talebi AA (2013) A study of the genus *Blacus* Nees (Hymenoptera: Braconidae: Blacinae) in Iran with three new records. Ukrainska Entomofaunistyka 4: 3–11.

[B14] Gosik R Sprick P Skuhrovec J Deruś M Hommes M (2016) Morphology and identification of the mature larvae of several species of the genus *Otiorhynchus* (Coleoptera, Curculionidae, Entiminae) from Central Europe with an update of the life history traits. Zootaxa 4108(1): 1–67. 10.11646/zootaxa.4108.1.1 27394846

[B15] Gosik R Sprick P Morris MG (2019) Descriptions of immature stages of four species of the genera *Graptus*, *Peritelus*, *Philopedon*, and *Tanymecus* and larval instar determination in *Tanymecus* (Coleoptera, Curculionidae, Entiminae). ZooKeys 813: 111–150. 10.3897/zookeys.813.30336 PMC6331514 30647529

[B16] Gosik R Skuhrovec J Caldara R Toševski I (2020) Immature stages of Palearctic *Mecinus* species (Coleoptera, Curculionidae, Curculioninae): Morphological characters diagnostic at genus and species levels. ZooKeys 939: 87–165. 10.3897/zookeys.939.50612 32577083 PMC7297811

[B17] Gosik R Wanat M Bidas M (2021) Adult Postabdomen, Immature Stages and Biology of *Euryommatusmariae* Roger, 1856 (Coleoptera: Curculionidae: Conoderinae), a Legendary Weevil in Europe. Insects 2(2): e151. 10.3390/insects12020151 PMC7916903 33670089

[B18] Hille Ris Lambers D (1950) On mounting aphids and other soft–skinned insects. Entomologische Berichten 13: 55–58.

[B19] Korotyaev BA Konstantinov AS O’Brien CW (2000) A new genus of Orobitidinae and discussion of its relationships (Coleoptera: Curculionidae). Proceedings of the Entomological Society of Washington 102: 929–956.

[B20] Kutzelnigg H (2013) Rekordverdächtige Konvergenzen. Beziehungen zwischen Pflanzen und Ameisen. Studium Integrale Journal 20(2): 76–83. http://www.si–journal.de/index2.php?artikel=jg20/heft2/sij202–2.html

[B21] Leibee GL Pass BC Yeargan KV (1980) Instar determination of Clover Root Curculio, *Sitonahispidulus* (Coleoptera: Curculionidae). Journal of the Kansas Entomological Society 53: 473–475. https://www.jstor.org/stable/pdf/25084061.pdf

[B22] Lohse GA (1983) 28. U.Fam. Ceutorhynchinae. In: Freude H Harde KW Lohse GA (Eds) Die Käfer Mitteleuropas.Band 11. Goecke & Evers, Krefeld, Germany, 180–253. [340 pp]

[B23] Lyal CHC (2013) Conoderinae. In: Smetana A (Ed.) Catalogus Coleopterorum Palearcticae, Curculionoidea II; Löbl I; vol.8. Brill, Leiden/Boston, 214–217.

[B24] Lyal CHC King T (1996) Elytro–tergal stridulation in weevils (Insecta: Coleoptera: Cyrculionoidea). Journal of Natural History 30(5): 703–773. 10.1080/00222939600770391

[B25] Marvaldi AE (1999) Morfología larval en Curculionidae. Acta Zoológica Lilloana 45: 7–24.

[B26] Marvaldi AE (2003) Key to larvae of the South American subfamilies of weevils (Coleoptera, Curculionoidea). Revista Chilena de Historia Natural 76(4): 603–612. 10.4067/S0716-078X2003000400005

[B27] May BM (1994) An introduction to the immature stages of Australian Curculionoidea. In: Zimmerman EC (Ed.) Australian weevils.Volume II: Brentidae, Eurhynchidae, Apionidae and a chapter on the immature stages by Brenda May. CSIRO Publications, Melbourne, Victoria, 365–728.

[B28] Morris MG (2012) True Weevils. (Coleoptera: Curculioninae, Baridinae, Orobitidinae). Part III. Royal Entomological Society of London. Handbook 5(17d): 1–246.

[B29] Nikulina ON (2013) New data on the larvae of the weevil tribe Baridini (Coleoptera, Curculionidae) from Mongolia and Middle Asia. Entomological Review 93(2): 199–207. 10.1134/S0013873813020085

[B30] Pakaluk J (1993) Review of the immature stages of Baridinae I: Nertinini (Coleoptera: Curculionidae). Elytron 7: 165–170.

[B31] Pakaluk J (1994) Review of the immature stages of Baridinae II: Madarini (Coleoptera: Curculionidae). Annales Zoologici 45: 1–14.

[B32] Pedroni G (2010) *Leiosomatalamellii* n. sp. della Majella (Appennino centrale) con alcune note di ecologia (InsectaColeopteraCurculionidae). Quaderno di Studi e Notizie di Storia Naturale della Romagna 30: 203–210.

[B33] Pedroni G (2012) Le specie italiane del gruppo di *Leiosomascrobiferum* con descrizione di sei specie nuove (Coleoptera, Curculionidae, Molytini). Bollettino del Museo Civico di Storia Naturale di Verona. Botanica, Zoologia 36: 73–90.

[B34] Pedroni G (2016) Designazione del lectotypus di *Leiosomakirschii* Gredler, 1866 (Coleoptera: Curculionidae: Molytinae). Gredleriana 16: 133–140.

[B35] Pedroni G (2018) Revisione tassonomica delle specie del gruppo di *Leiosomacribrum* (Coleoptera: Curculionidae: Molytinae).- Atti della Società italiana di scienze naturali e del Museo civico di storia naturale di Milano 5(1): 19–32.

[B36] Penecke KA (1922) Beiträge zur Kenntnis der geographischen Verbreitung und der Nährpflanzen von Curculioniden. Wiener Entomologische Zeitung 39(5–10): 183–188. 10.5962/bhl.part.2574

[B37] Prena J Colonnelli E Hespenheide HA (2014) Conoderinae Schoenherr, 1833. In: Leschen RAB Beutel RG (Eds) Handbook of Zoology, Coleoptera, vol.3. DeGruyter Berlin, 577–589.

[B38] Rowe DJ Kok LT (1985) Determination of larval instars, and comparison of field and artificial diet–reared larval stages of *Rhinocyllusconicus* (Col: Curculionidae). Virginia Journal of Science 36: 277–280.

[B39] Sabaljuev IA (2013) Dolgonosik [Дoлгoнocик] *Leiosomareitteri* Bedel, 1884. https://www.zin.ru/animalia/coleoptera/rus/leireiza.htm [accessed on 26 April 2022]

[B40] Scherf H (1964) Die Entwicklungsstadien der mitteleuropäischen Curculioniden (Morphologie, Bionomie, Ökologie). Abhandlungen der Senckenbergischen Naturforschenden Gesellschaft 506: 1–335.

[B41] Skuhrovec J Gosik R Caldara R Košťál M (2015) Immatures of Palaearctic species of the weevil genus *Sibinia* (Coleoptera, Curculionidae): New descriptions and new bionomic data with suggestions on their potential value in a phylogenetic reconstruction of the genus. Zootaxa 3955: 151–187. 10.11646/zootaxa.3955.2.1 25947846

[B42] Skuhrovec J Gosik R Caldara R Toševski I Batyra A (2022) Description of immature stages of *Gymnetron* species (Coleoptera, Curculionidae, Curculioninae), with particular emphasis on the diagnostic morphological characters at the generic and specific levels. ZooKeys 1090: 45–84. 10.3897/zookeys.1090.78741 35586838 PMC8967815

[B43] Smreczyński S (1974) Chrząszcze – Coleoptera. Ryjkowce – Curculionidae. Podrodzina Curculioninae. Plemiona Barini, Coryssomerini, Ceutorhynchini, vol. 83 (XIX, 98e). Klucze do Oznaczania Owadów Polski, Warszawa, 180 pp.

[B44] Sprick P Gosik R (2014) Biology and morphology of the mature larva of *Mitoplinthuscaliginosuscaliginous* (Curculionidae, Molytinae). SNUDEBILLER: Studies on taxonomy, biology and ecology of Curculionoidea 15, 229, 10 pp.

[B45] Sprick P Krämer-Klement K (2022[in press]) Oxalidaceae, eine neue Wirtspflanzenfamilie für die Gattung *Leiosoma* Stephens, 1829 - mit Bemerkungen zu den Wirtspflanzen von *Leiosoma*-Arten (Curculionidae: Molytinae). Weevil News.

[B46] Sprick P Gosik R Graba S (2022) Morphology, instar determination and larval biology of Otiorhynchus (Otiorhynchus) coecus coecus Germar, 1823 and pupal morphology of O. (Nihus) carinatopunctatus (Retzius, 1783) and O. (Postaremus) nodosus (O. F. Müller, 1764) from Central and Northern Europe (Coleoptera, Curculionidae, Entiminae). Weevil News 99: 1–24.

[B47] Stüben PE (2011) Die Curculionoidea (Coleoptera) La Gomeras. Snudebiller : Studies on Taxonomy, Biology, and Ecology of Curculionoidea 12(177): 85–129.

[B48] Urban C (1925) Der Veilchenkäfer. Entomologische Blätter 21: 139–141.

[B49] Van Emden FI (1938) On the taxonomy of Rhynchophora larvae (Coleoptera). The Transactions of the Entomological Society of London 8(1): 1–37. 10.1111/j.1365-2311.1938.tb01800.x

[B50] Willis RJ (1964) The bionomics and larval morphology of the otiorrhynchid pests of soft fruit crops.– Thesis, The Queen’s University of Belfast, Northern Ireland, 250 pp. [and appendix of 60 pp.]

[B51] Zherikhin VV Gratshev VG (1995) A comparative study of the hind wing venation of the superfamily Curculionoidea, with phylogenetic implications. In: Pakaluk J Ślipiński SA (Eds) Biology, phylogeny and classification of Coleoptera: Papers celebrating the 80th birthday of Roy A.Crowson. Vol. 2. Muzeum i Instytut Zoologii PAN, Warsaw, 633–777. [i–vi + 559–1092 pp]

